# Telomere elongation in parthenogenetic stem cells

**DOI:** 10.1007/s13238-013-0006-z

**Published:** 2014-01-31

**Authors:** Yu Yin, Na Liu, Xiaoying Ye, Renpeng Guo, Jie Hao, Fang Wang, Lin Liu

**Affiliations:** State Key Laboratory of Medicinal Chemical Biology, Department of Cell Biology and Genetics, College of Life Sciences, Nankai University, Tianjin, 300071 China

**Dear Editor,**

Parthenogenetic embryonic stem (pES) cells, generated from oocytes by artificial activation without involvement of fertilization, show differentiation and pluripotency as evidenced by their capacity to generate germline chimeras and all pES pups by tetraploid embryo complementation, indicating the ability of pES cells to form all cell types in the body (Chen et al., 2009; Liu et al., 2011). Indeed, pES cells can repair injured muscle (Koh et al., 2009) and cardiomyocytes with reduced risk of tumorigenesis (Liu et al., 2013), and contribute to long-term hematopoiesis (Eckardt et al., 2007), supporting the potential applications of pES cells in cell transplantation therapy and tissue engineering (Koh et al., 2009). Furthermore, successful derivation of human pES cells provides important pluripotent stem cell sources alternative to ES cells (or fES, ES cells derived from fertilized embryos) for future clinic therapeutic uses (Mai et al., 2007). Telomere length maintenance is critical for genomic stability, unlimited self-renewal, and developmental pluripotency of ES cells. It remains elusive whether telomeres are sufficiently reprogrammed in pES cells.

We thought to analyze the telomere lengths of pES cells, characteristic of ES cells in morphology (Fig. 1A), in comparison with those of ES cells at similar passages. ES and pES cells were depleted off mouse embryonic fibroblasts (MEF) as feeder prior to harvest for analysis in subsequent experiments. We show that telomeres elongated, and were even slightly longer in pES than in ES cells. Two different pES cell lines (C3 and 1116) exhibited longer telomeres than did ES cells with identical genetic background estimated initially by telomere qPCR analysis (Fig. 1B), and also by quantitative telomere FISH (QFISH) method (Fig. 1C and 1D). Moreover, telomeres of pES cells elongated slightly during passages, like those of ES cells (BF10). The telomere QFISH data were generally consistent with relative telomere length expressed as T/S ratio by qPCR. Also, two other pES cell lines generated from oocytes of inbred young C57BL/6 mice displayed telomere maintenance or elongation during passages, like fES cells (N33) (Fig. 1E). Together, telomeres are reprogrammed and sufficiently elongated in pES cells.

**Figure 1:**
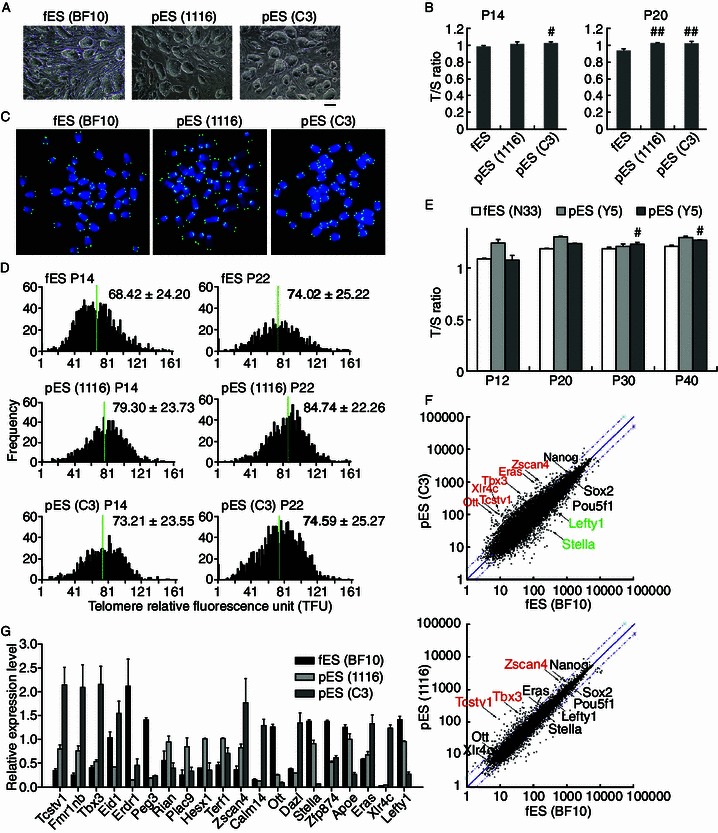
Telomere length and genome-wide gene expression of pES cells versus ES (fES) cells. (A) Colony morphology of pES cells (1116, C3) and fES cells (BF10) at passages 13–15. (B) Relative telomere length expressed as T/S ratio measured by quantitative real-time PCR method. Error bars indicate mean ± SD (at least two repeats). *, *P* < 0.05; **, *P* < 0.01, compared to fES cells. (C) Telomere quantitative FISH images of chromosome spread from pES and fES cells. Green dots, telomeres; blue, DAPI-stained chromosomes. (D) Distribution histogram showing relative telomere length (TFU) of pES cells and fES cells, analyzed by telomere Q-FISH and TFL-TELO software (10–15 spreads analyzed for each cell line). (E) Longer telomere length expressed as T/S ratio estimated by qPCR in pES (Y5 and Y6) derived from oocytes of C57BL/6 mice, compared with fES (N33) from the same genetic background. *, *P* < 0.05, compared to the corresponding pES at P12. (F) Scatter plot showing comparison of global gene expression of pES cells and fES cells. Genes up-regulated (highlighted in red) and down-regulated (in green) in pES cells (C3 and 1116) were compared with those of fES cells. Genes are listed in Tables S1 and S2 using cut off as fold ≥ 2.0. (G) Real-time PCR validation of selected 20 genes differentially expressed between pES and fES cells by microarray

To investigate the molecular bases of differential telomere elongation, we performed global gene expression analysis of pES cells, compared with fES cells by microarray. Genes important for development and differentiation showed no or only minimal differences in their expression between pES and fES cells, and were not enriched in the differentially expressed gene lists (Tables S1 and S2). Expression of genes associated with pluripotency of ES cells, such as *Pou5f1* (*Oct4*), *Nanog*, *Sox2*, and *Rex1* did not differ among these three cell lines. Major telomerase genes *Tert* and *Terc* also did not show differential expression between pES and ES cells.

Interestingly, most of the up-regulated genes in both pES cell lines were enriched in 2-cell embryo state, including *Tcstv1/3, Dub1, Eif1α*, *Gm4340*, and *Zscan4* (Zalzman et al., 2010; Macfarlan et al., 2012). Differential gene expression profile also was found between two pES cell lines, but pES cells 1116 closely resembled ES cells more than did pES cells C3 (Fig. 1F). For instance, *Lefty1* and *Stella* (also known as *Dppa3*), expressed at reduced levels in pES cells C3, compared with pES 1116 or ES cells. Coincidently, chimeras generated from pES 1116 but not C3 showed germline competency (Liu et al., 2011). The microarray data were validated by qPCR analysis of selected genes, although the fold in relative expression levels showed some differences for a few genes (Fig. 1G).

By immunofluorescence microscopy, *Zscan4* was expressed sporadically in only small proportion (1%–5%) of ES cell cultures, consistent with the reports (Zalzman et al., 2010; Macfarlan et al., 2012). While some of Zscan4 positive ES cells were excluded from Oct4 expression, a few others showed weak positive staining for Oct4 (Fig. 2A). In addition, the proportion of *Zscan4* positive cells was increased in pES cells, with higher ratio in pES C3 by both flow cytometry and immunofluorescence microscopy quantification (Fig. 2A–C). The protein levels of Zscan4 also were higher in pES cells than in ES cells, and highest in pES cell C3 (Fig. 2D), consistent with flow cytometry and immunofluorescence quantification data. Moreover, murine endogenous virus element (MERV) expressed at higher levels in pES than in ES cells by qPCR (Fig. S1A and S1B).

**Figure 2:**
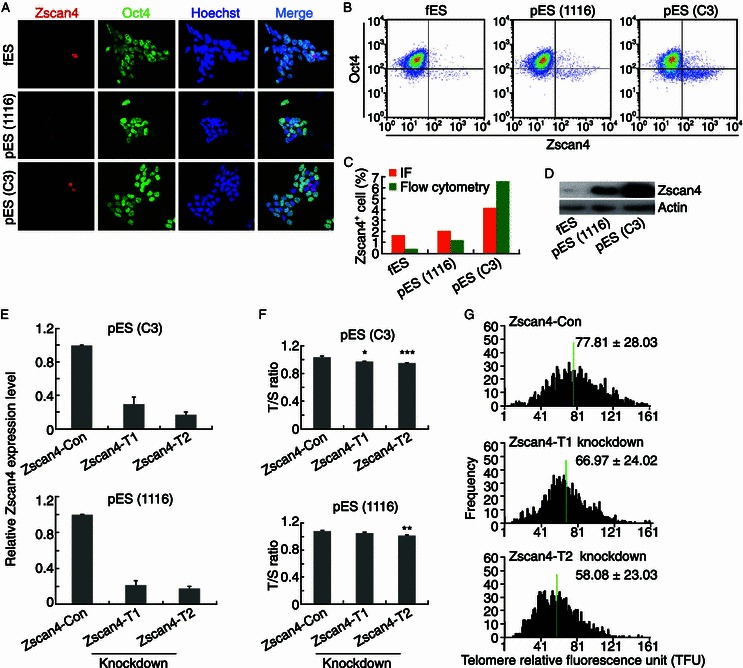
Involvement of *Zscan4* in telomere elongation of pES cells. (A) Immunofluorescence images of Zscan4 (red) and Oct4 (green) in pES (1116 and C3) versus fES cells (BF10) at passage 15–16. Nuclei stained with Hoechst 33342 (blue). (B) Analysis of Zscan4^+^ cells in pES or fES cell cultures at passage 15–16 by flow cytometry. Two repeats. (C) Proportion of Zscan4^+^ cells in pES cell cultures (1116 and C3) compared with fES cell cultures (BF10) by flow cytometry and immunofluorescence microscopy. (D) Protein levels of Zscan4 in fES cells (BF10) and pES cells (1116 and C3) at passage 15–16 by Western blot. β-Actin served as loading control. (E) *Zscan4* expression levels by qPCR are notably lower in *Zscan4* stable knockdown (KD) pES cells (C3 or 1116) by two shRNAs (T1, T2), compared with knockdown controls. (F) Telomeres of *Zscan4* knockdown (Zscan4-T1 and Zscan4-T2) pES cells (C3 or 1116) are shorter than those of control knockdown pES cells (Zscan4-Con) at passage 20 analyzed by qPCR. Error bars indicate mean ± SD. *, *P* < 0.05; **, *P* < 0.01; ***, *P* < 0.001, compared with control knockdown. (G) Distribution histogram showing relative telomere length as TFU in *Zscan4* knockdown (Zscan4-T1 and Zscan4-T2) pES cells (C3) and in control knockdown cells (Zscan4-Con) at passage 20, analyzed by telomere Q-FISH and the TFL-TELO software. Medium telomere lengths are indicated by green bar

*Zscan4* was shown to lengthen telomeres of ES cells presumably via recombination based mechanism (Zalzman et al., 2010). To examine whether elevated levels of *Zscan4* also are implicated in telomere elongation of pES cells, we knocked down *Zscan4* in two pES cell lines by two independent shRNAs effectively targeting *Zscan*4 (Fig. 2E). Depletion of *Zscan4* shortened telomeres in both pES cell lines estimated by QFISH as well as qPCR (Fig. 2F and 2G), suggesting that *Zscan4* might involve in telomere elongation of pES cells. However, various telomere lengths of pES cells (Fig. 1B–D), did not completely correlate with absolute Zscan4 protein levels (Fig. 2D), suggesting that genes other than *Zscan4* may also play roles in telomere elongation and self-renewal of pES cells.

Moreover, epigenetic modifications at telomeres and subtelomeres regulate telomere lengths (Blasco, 2007). Active histones H3K4me3, H3K9Ac, H3Ac and repressive histone H3K27me3 mostly enriched in euchromatin did not show noticeable differences in their protein levels between pES and ES cells, whereas heterochromatic repressive H3K9me3 levels were reduced in pES cells compared with ES cells (Fig. S1C). Lower levels of H3K9me3 may de-repress *Zscan4* and *Tcstv1* located at subtelomeres.

Reduced MAPK and increased Wnt Signaling are implicated in derivation, self-renewal, and pluripotent state of mouse ES cells. Parthenogenetic ‘blastocysts’ display reduced MAPK and increased Wnt signaling (Liu et al., 2010) and this may contribute to more efficient derivation of pES cells in mice (Chen et al., 2009) as well as in human (Mai et al., 2007). Membrane β-catenin was found in both pES and ES cells (Fig. S2A), and nuclear β-catenin protein expressed at relatively higher levels in pES than in ES cells (Fig. S2B), although total β-catenin levels seemed not to differ between the two cell lines (Fig. S2C). Erk levels appeared slightly lower in pES 1116, relative to ES cells, but much lower in pES C3 (Fig. S2C), which showed highest Zscan4 levels.

Factors in oocytes or early cleavage embryos presumably contribute to both rapid telomere reprogramming and epigenetic reprogramming during early embryo development. These critical factors can be exploited to improve reprogramming induction of iPS cells. Notably, telomeres elongate slowly during iPS cell induction and gradually acquire ES cell telomere lengths during continued passages (Wang et al., 2012), different from rapid telomere lengthening in early cleavage embryos (Liu et al., 2007). Our data suggest that increased expression of 2-cell genes particularly *Zscan4* may partially explain telomere elongation of pES cells. Also, *Zscan*4 proves to rapidly lengthen telomeres and greatly enhance genomic stability and quality of iPS cells (Jiang et al., 2013). Multiple factors can influence pluripotency and germline competency of ES/iPS/pES cells. The functional significance of telomere elongation in pES cells or slightly longer telomeres in pES cells relative to ES cells remains unclear, but speculatively the robust telomere maintenance may help maintenance of self-renewal of pES cells *in vitro*.

In summary, pES cells generated from parthenogenetically activated oocytes exhibit telomere elongation or even slightly longer telomeres compared with fES cells. Without complication of sperm factors, parthenogenetically activated oocytes themselves can effectively elongate telomeres during early cleavage development (Liu et al., 2007). Consistently, somatic cell nuclear transfer (SCNT) using oocytes further improves chromatin remodeling and telomere elongation of iPS cells (Liu et al., 2012). SCNT efficiency in cloning human embryos has been remarkably improved recently with success at long last, such that human SCNT ES cells now are effectively achieved (Tachibana et al., 2013). It would be interesting to further explore whether telomeres are effectively elongated to maintain genomic stability of SCNT ES cells achieved using oocyte factors.

## Electronic supplementary material

Below is the link to the electronic supplementary material.

Supplementary material 1 (PDF 180 kb)

Supplementary material 2 (XLS 352 kb)

Supplementary material 3 (XLS 55 kb)
