# Supplementary material for: Telomere elongation in parthenogenetic stem cells
Source: Protein Cell. 2014 Jan 31;5(1):8–11. doi: 10.1007/s13238-013-0006-z (PMC3938844; doi:10.1007/s13238-013-0006-z)
Supplement: Supplementary file 1 — Supplementary material 1 (PDF 180 kb) [file 13238_2013_6_MOESM1_ESM.pdf]

Supplemental Materials

Telomere elongation in parthenogenetic stem cells

Yu Yin, Na Liu, Xiaoying Ye, Renpeng Guo, Jie Hao, Fang Wang, Lin Liu

Supplemental Methods
Supplemental Tables 1-3
Supplemental Figures S1-2

Methods

ES and pES cells and cell culture

ES cell lines (F1 or BF10 derived from C57BL/6XC3H, N33 from C57BL/6, NF2 from Nanog-EGFP transgenic mice), and pES cell lines (C3 and 1116 derived from C57BL/6xC3H, Y5 and Y6 from C57BL/6, Ivm4 from Nanog-EGFP transgenic mice) were isolated and characterized as described previously, and cultured under standard ES cell culture conditions (Chen et al., 2009; Huang et al., 2010; Liu et al., 2011). ES cell medium is composed of knockout Dulbecco's modified Eagle medium added with 20% fetal bovine serum, 1000 U/ml LIF, 0.1 mM β-mercaptoethanol, 1 mM L-glutamine, 0.1 mM nonessential amino acids, 100 units/ml penicillin and 100 μg/ml streptomycin.

Microarray

ES (BF10) cells and pES cells (C3 and 1116) were collected for global gene expression analysis using Affymetrix mouse genome 430 2.0 array. Hybridized arrays were scanned with a Gene Array Scanner (Affymetrix). Signal intensities were calculated and array comparisons performed with MAS 5.0. Only probe sets showing two fold changes in duplicate experiments were retained in the final list. The detection call, indicating whether a transcript was reliably detected (P, Present) or not (A, Absent), was also used as a parameter in obtaining the final list. Hierarchical clustering was performed with the above differentially expressed genes using cluster software (version 3) and by applied

mean centering and normalization of genes and arrays before average linkage clustering.

Gene expression analysis by quantitative real-time PCR

Total RNA was isolated using TRIzol Reagent (Invitrogen). 2μg of RNA was subject to cDNA synthesis using M-MLV Reverse Transcriptase (Invitrogen). PCR reaction was set up in duplicates using the FastStart Universal SYBR Green Master (ROX, Roche) and run on the iCycler real-time PCR detection system (Bio-Rad) using primer sets specific for each gene (Supplementary Table 3).

Flow cytomety analysis

Zscan4 positive ES cells were analyzed by fluorescence activated cell sorting (FACS) using BD FACSAria. The primary antibody anti-Zscan4 (#5114, Custom made, GenScript) and anti-Oct 4 (sc5279, Santa Cruz) and secondary antibody (Goat anti Mouse IgG (H+L), FITC, 115-095-003, Jackson; Goat anti Rabbit IgG (H+L), Alexa Fluor® 594, 111-585-003, Jackson) were used in this analysis.

Immunofluorescence microscopy

Cells were washed twice in phosphate-buffered saline (PBS), then fixed in freshly prepared 3.7% paraformaldehyde in PBS (pH 7.4), permeabilized in 0.1% Triton X-100 in blocking solution (3% goat serum in PBS) for 30 min, washed and left in blocking solution for 1 h. Cells were incubated overnight at 4 ºC with primary antibodies against Oct4 (sc5279, Santa Cruz), Zscan4 (#5114, Custom made, GenScript), or β-catenin (ab16051, Abcam), washed and incubated for 1 h with appropriate secondary antibodies (Goat anti Mouse IgG (H+L), FITC, 115-095-003, Jackson; Goat anti Rabbit IgG (H+L), Alexa Fluor® 594, 111-585-003, Jackson). Samples were counterstained with 0.5μg/ml Hoechst 33342 in Vectashield mounting medium. Fluorescence was imaged using a Zeiss fluorescence microscope.

Western Blot

Cells were collected and washed with cold PBS, then resuspended in cell lysis buffer containing 50 mM Tris (pH 7.4), 150 mM NaCl, 1 mM EDTA, 1 mM EGTA, 1 mM NaF, 20 mM Na4P2O7, 1 mM Na3VO4, 1%Triton X-100, 10% glycerol, 0.25% deoxycholate and 0.1% SDS. 20 μg of proteins were separated on 10% SDS-polyacrylamide gels and transferred to polyvinylidene difluoride (PVDF, Millipore) membranes. Nonspecific binding was blocked by incubation in 5% skim milk in TBST overnight at 4ºC. Blots were then probed for 2-4 hours at room temperature with primary antibodies against Zscan4 (#5114, Custom made, GenScript), β-catenin (ab16051, Abcam), pErk (sc7383, Santa Cruz), Erk (sc-94, Santa Cruz), H3 (ab1791, Abcam), β-Actin (sc1616R, Santa Cruz), H3K4me3 (ab1012, Abcam), H3K9me3 (07-442, Millipore), H3K27me3 (07-449, Millipore), H3Ac (06-599, Millipore), H3K9Ac (07-352, Millipore), washed and incubated for 2 hours at room temperature with secondary antibodies HRP conjugated donkey anti-Rabbit IgG (GE Healthcare NA934v) or goat anti-mouse IgG (H+L) (ZB2305). Protein bands were detected using ECL western blotting detection reagent (Millipore WBKLS0100).

For nuclear protein extraction, 2×10^6 cells were harvest and treated with 200 μl ice-cold lysis buffer A (10 mM Hepes-NaOH (pH 7.9), 10 mM KCl, 1.5 mM $MgCl_2$, added with 1,0 mM protease inhibitor cocktail and 1.0 mM PMSF prior to use) and incubated on ice for 15 min, then added with 10 μl buffer B (10% NP-40), vortex and centrifuged. The supernatant was discarded and the pellet washed with ice-cold buffer A and add 50 μl nuclei lysis buffer C (20 mM Hepes-NaOH (pH 7.9), 420 mM NaCl, 3 mM EDTA, 1 mM DTT 2mM $MgCl_2$, 25% Glycerol, added with 1,0 mM protease inhibitor cocktail and 1.0 mM PMSF prior to use). The pellet dispersed and put on ice for 30 min, vortex for 15 s every 3 min, centrifuged at 13000g for 15 min, and the nuclear proteins were extracted.

Zscan4 RNAi

For stable knockdown of Zscan4, shRNA sequences (Zalzman et al., 2010) were synthesized and cloned into pSIREN-retroQ RNAi vector according to manufacturer's instructions. The negative control shRNA without sequence homology to mouse genes served as negative control. The RNAi retrovirus was packaged using Plat-E cells and then infected pES cells. 48h after infection, the cells were selected using 2 μg/ml puromycin for 7 days, and clones were picked.

Telomere QFISH

Telomere length and function (telomere integrity and chromosome stability) was estimated by telomere quantitative FISH. Cells were incubated with 0.5 μg/ml nocodazole for 1.5 h to enrich cells at metaphases (Liu et al., 2007). Chromosome spreads were made by standard method. Metaphase-enriched cells were exposed to hypotonic treatment with 75 mM KCl solution, fixed with methanol: glacial acetic acid (3:1) and spread onto clean slides. FITC-labeled (CCCTAA) peptide nucleic acid (PNA) probe was used in this study. Telomeres were denatured at 80 ºC for 3 min and hybridized with telomere PNA probe (0.5μg/ml) (Panagene, Korea). Chromosomes were stained with 0.5μg/ml DAPI. Fluorescence from chromosomes and telomeres was digitally imaged on a Zeiss microscope with fluorescein isothiocyanate (FITC)/DAPI filters, using AxioCam and AxioVision software 4.6. Telomere length shown as telomere fluorescence intensity was integrated using the TFL-TELO program (a gift kindly provided by Peter Lansdorp, Terry Fox Laboratory).

Telomere measurement by quantitative real-time PCR

Cells were washed in PBS and stored at -20ºC until subsequent DNA extraction. Genome DNA was prepared using DNeasy Blood & Tissue Kit (Qiagen, Valencia, CA). Average telomere length was measured using real-time PCR assay, as previously described, but

modified for measurement of mouse telomeres (Liu et al., 2007). PCR reactions were performed using the iCycler iQ real-time PCR detection system (Bio-Rad), using telomeric primers, primers for the reference control gene (mouse 36B4 single copy gene). For each PCR reaction, a standard curve was made by serial dilutions of known amounts of DNA. The telomere signal was normalized to the signal from the single copy gene to generate a T/S ratio indicative of relative telomere length. Equal amounts of DNA were used for each reaction.

Statistics

Data were analyzed by analysis of variance and means compared by Fisher's protected least-significant difference using the StatView software from SAS Institute Inc. (Cary, NC), a value of $P<0.05$ was considered statistical significance.

Supplemental Table S1. Differentially expressed genes between pES C3 and fES BF10.xls

Supplemental Table S2. Differentially expressed genes between pES 1116 and fES BF10.xls

Supplemental Table S3. Primers for qPCR analysis of related genes

Genes	Forward	Reverse
β-actin	GAGATTACTGCTCTGGCTCCTA	GGACTCATCGTACTCCTGCTTG
Tbx3	CCACCCGTTCCTCAATTTGAACAG	CGGAAGCCATTGATGGTAAAGCTG
Zscan4	AAATGCCTTATGTCTGTTCCCTATG	TGTGGTAATTCCTCAGGTGACGAT
Tcstv1	TGAACCCTGATGCCTGCTAAGACT	AGATGGCTGCAAAGACACAACTGC
Ott	ATGGCGAACCATGAAGACGAA	GTCCAGGGAAAAATGCTTGCC
Dazl	ATGTCTGCCACAACTTCTGAG	CTGATTTCGGTTTCATCCATCCT
Plac9	GTGCAAAGGCGGTTAGACATT	GCCCTGTGGGAAGGTTTGA
Terf1	TGACAGCGCCGAGGCTATTATTCA	AAACTGTGCATCAAGGGCCTTTCC
Lefty1	CCAACCGCACTGCCCTTAT	CGCGAAACGAACCAACTTGT
Apoe	CTGACAGGATGCCTAGCCG	CGCAGGTAATCCCAGAAGC
Zfp874	AAAACCAAGGATCTTCGGGTG	GGCATTGCATACTTAGAGAGCTG
Calml4	TCTTCTGGTGTCCATGAGGTG	GAAGTCCAGCTCTCCGTTCTT
Peg3	TCATGCACACTAGGGAGAACC	GGCAGCACTCCTACTGAAGG
Eras	TGCCTACAAAGTCTAGCATCTTG	CTTTTACCAACACCACTTGCAC
Eid1	CGGCGAGGGCTATATGGAG	TTCAGGGAACTCGTAGTCGTC
Hesx1	TGAGAGCATTTTAGGACTGGACC	GGGTGGGTTGCCATCTTTCT
Fmr1nb	TCCTGGGATTTCTGCCTATGTGCT	ACGCCTTCCTTCCTTTAGGCTTCT
Erdr1	ACTCACCACAGCACACAGGACACA	CGTTAGCACATACATCTTGACCGC
Xlr4c-M1	TGGTGAGTACAGCTCTCAAACGGA	AGGGCCAGATTGTTTCCTGGGTTA
Gapdh	TCAACAGCAACTCCCACTCTTCCA	ACCACCCTGTTGCTGTAGCCGTAT
MERVL1	CCCATCATGAGCTGGGTACT	CGTGCAGATCCATCAGTAAA
MERVL2	CCCATCATGAGCTGGGTACT	CGTGCAGAGCCATCAGTAA
MulV	GGCGCCCCGTACAAGATTTCATA	GATAACGGGCCTGCCTTCACCTC
Line1	TTTGGGACACAATGAAAGCA	CTGCCGTCTACTCCTCTTGG
SineB1	GTGGCGCACGCCTTTAATC	GACAGGGTTTCTCTGTGTAG
MusD	GATTGGTGGAAGTTTAGCTAGCAT	TAGCATTCTCATAAGCCAATTGCAT
IAP-Gag	AATCTCAGAACCGCTCCATGA	TTTCTTAAAATGCCCAGGCTTT

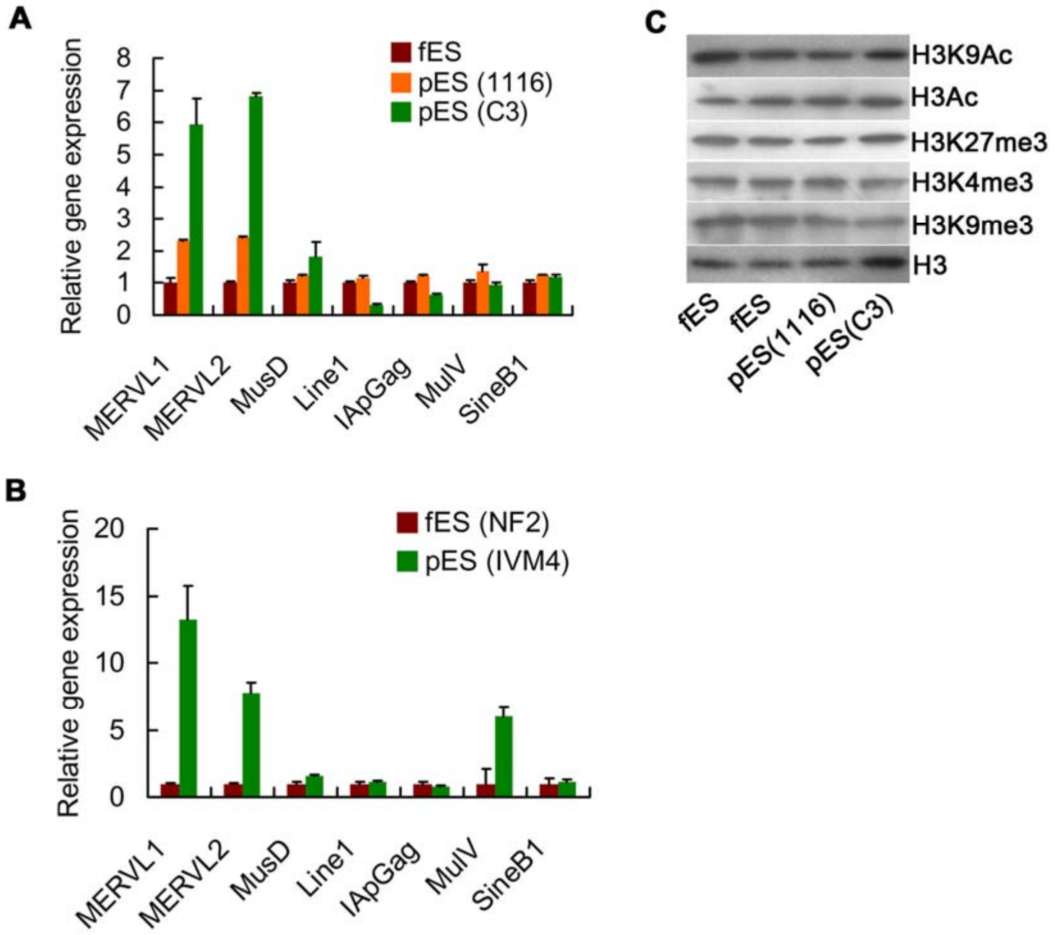

Supplemental Fig. S1. pES cells express higher levels of endogenous retroviruses including *MERVL* than those of fES cells at passage 14. **(A)** pES 1116 and C3 vs fES BF10. **(B)** pES IVM4 vs fES NF2 from activated Nanog-GFP mouse oocytes. **(C)** Levels of histone modifications in fES and pES at similar passages by Western blot analysis.

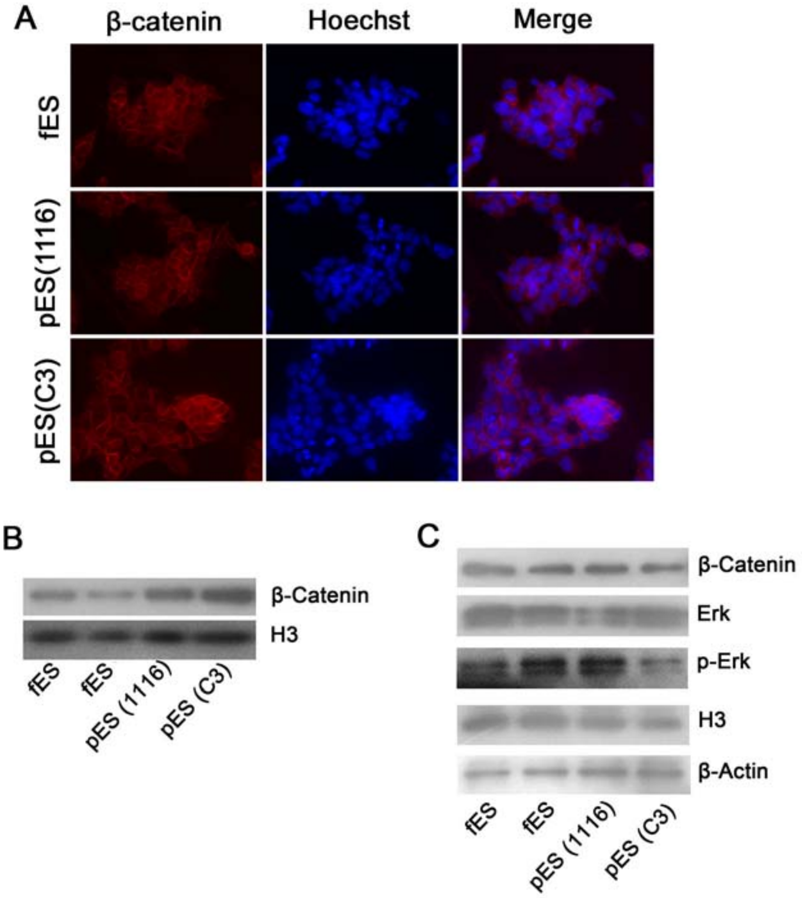

Supplementa. Fig. S2. Comparison of Wnt and MAPK signaling between pES and fES cells. **(A)** Immunofluorescence staining of ß-catenin (red) in fES cells (BF10) and pES cells (1116 and C3) at passage 15~16. Nuclei stained with Hoechst 33342 (blue). **(B)** Protein levels of nuclear ß-catenin in fES cells (BF10) and pES cells (1116 and C3). Nuclear extracts were prepared for the analysis at passage 15~16. H3 served as loading control. **(C)** Protein levels of total ß-catenin, Erk and p-Erk in fES cells (BF10) and pES cells (1116 and C3) at passage 15~16. Whole cell lysis was used for the western-blot analysis. ß-Actin served as loading control.

Supplemental References

Chen, Z., Liu, Z., Huang, J., Amano, T., Li, C., Cao, S., Wu, C., Liu, B., Zhou, L., Carter, M.G., *et al.* (2009). Birth of parthenote mice directly from parthenogenetic embryonic stem cells. Stem Cells 27, 2136-2145.

Huang, J., Okuka, M., Wang, F., Zuo, B., Liang, P., Kalmbach, K., Liu, L., and Keefe, D.L. (2010). Generation of pluripotent stem cells from eggs of aging mice. Aging Cell 9, 113-125.

Liu, L., Bailey, S.M., Okuka, M., Munoz, P., Li, C., Zhou, L., Wu, C., Czerwiec, E., Sandler, L., Seyfang, A., *et al.* (2007). Telomere lengthening early in development. Nat Cell Biol 9, 1436-1441.

Liu, Z., Hu, Z., Pan, X., Li, M., Togun, T.A., Tuck, D., Pelizzola, M., Huang, J., Ye, X., Yin, Y., *et al.* (2011). Germline competency of parthenogenetic embryonic stem cells from immature oocytes of adult mouse ovary. Hum Mol Genet 20, 1339-1352.

Zalzman, M., Falco, G., Sharova, L.V., Nishiyama, A., Thomas, M., Lee, S.L., Stagg, C.A., Hoang, H.G., Yang, H.T., Indig, F.E., *et al.* (2010). Zscan4 regulates telomere elongation and genomic stability in ES cells. Nature 464, 858-863.
